# "The Hospital" Nursing Mirror

**Published:** 1899-09-16

**Authors:** 


### "The Hospital" Nursing Mirror.

Being the Nursing Section of "The Hospital."

[Contributions for this Section of "The Hospital" should be addressed to the Editor, The Hospital, 28 & 29, Southampton Street, Strand, London, W.C., and should have the word "Nursing" plainly written in left-hand top corner of the envelope.]

### Notes on News from the Nursing World.

### OUR CHRISTMAS DISTRIBUTION.

The numerous friends who are kind enough to enable us year by year to make a distribution at Christmas of warm and useful gifts among the patients in the hospitals and infirmaries will pardon us for reminding them that it is none too soon to commence work. They will share our desire that the articles contributed should not only be serviceable, but numerous. The more liberally they respond to an appeal the more help they will afford to the nurses who so joyfully charge themselves with the task of opening the parcels and distributing the garments, and the more pleasure they will give to the sick and suffering at a season of the year when the wants of others should be the special consideration of us all.

### NURSING IN IRISH WORKHOUSES.

The conditions of nursing in Irish workhouses are exciting a good deal of controversy just now, and the question of the necessary qualifications seems in some doubt among the members of Irish Boards of Guardians. We accordingly wrote to the Secretary of the Irish Local Government Board for official information, and we inquired particularly the conditions on which nuns are admitted to workhouses as nurses. After a considerable interval we received from Mr. H. M. Swaine, the Acting Assistant Secretary, a courteous reply, enclosing a copy of the circular letter of January 12th last, prescribing the qualifications necessary in the case of any person claiming to be a "trained nurse" for the purpose of the Act of 1898. No reference was made to the inquiry about nuns. Here, however, is the substance of the circular issued by the Local Government Board:—

"The Local Government Board have to inform you that it is proposed, in due course, to prescribe the following qualifications as necessary in the case of any person claiming to be a 'trained nurse' for the purposes of section 58, sub-section 2 (*a*) (ii.) of the Local Government (Ireland) Act, 1898:—

> "'The term "trained nurse" shall mean any person who has resided for not less than two years in a clinical or other hospital recognised by the Local Government Board, and who, after examination, has obtained from such hospital a certificate of proficiency in nursing.'

"The Board have to point out, in connection with this matter, that they will not be prepared to accept the certificate of the authorities and staff of any hospital, except a clinical hospital recognised by the medical examining bodies in Ireland, England, or Scotland, unless the non-clinical hospital has at least 150 beds for medical and surgical cases, and unless due provision is also made for giving the probationer nurses a course of training in the nursing of cases of infectious diseases. In addition, the hospital should have a staff of at least one resident and two visiting physicians and a trained head nurse. Such arrangements should likewise be made, including the giving of lectures and the holding of examinations, as shall satisfy the Local Government Board that sufficient opportunities are afforded to the persons undergoing instruction to become fully trained, experienced, and certificated nurses."

We may have more to say respecting this subject on a future occasion.

### THE LATE NURSE MURPHY.

The latest victim of plague in the nursing profession is Miss Maud Murphy. The news of her death at Belgaum, Bombay, was received by cable at the India Office last week. Belgaum is a city over a couple of hundred miles from Poonah, and is on the same line of railway communication. It is hardly surprising, therefore, that the pestilence which raged at Poonah should have made its appearance there. The returns at the India Office show a mortality during the outbreak of 1898 of nearly 300 a week; whilst the same register records no less than 600 deaths per week during the present epidemic. Miss Murphy went out as a plague nurse in January, 1898, and having emerged from the first year's ordeal safe and sound, she stayed on for a further term of service. We are indebted to the matron of her training school, St. Pancras Infirmary, Dartmouth Park Hill, for an outline of her career. Nurse Maud Murphy entered the infirmary for training on May 3rd, 1887. She was promoted to be charge nurse of one of the female wards, and left on January 26th, 1893, to take up private nursing. She afterwards took an appointment as charge nurse at the North-Western Fever Hospital, but had to give it up owing to a severe attack of diphtheria, which obliged her to take a long rest. In May, 1895, she went to the National Hospital for the Paralysed and Epileptic, Queen's Square, as charge nurse of a male ward; and in December, 1896, she was made sister of the ward. In January, 1898, she proceeded to Bombay for plague work. She was a very bright, quick probationer, and when she became charge nurse she was very kind and attentive to her patients. In fact, she was one of the most skilful and efficient nurses the matron of St. Pancras Infirmary ever knew. She was also very obliging to all with whom she worked. The matron adds: "She always kept up her old feeling for our infirmary, and came to us whenever she could. I need not tell you how deeply we feel her loss. Personally, I was much attached to her, and feel her death keenly." This testimony to a sweet, unselfish life will accentuate the regret that it has been cut short by pneumonic plague. Yet, the sad fate of Miss Murphy will not prevent nurses from volunteering for the five vacancies which have to be filled up on the active staff now in India, nor deter them from adding their names to the reserve list at the India Office.

### THE AUCKLAND DISTRICT COTTAGE HOSPITAL.

Among the goodly company who attended the ceremony of opening the Auckland District Cottage Hospital by Lord Rosebery last Friday were many nurses. Curiously enough, in his interesting speech on the occasion, Lord Rosebery made no reference to nursing. But he gratified nurses by the graceful compliments he paid to Lady Eden, after whom the new hospital is called. She was the initiator of the building, and it is mainly through her efforts that it has been erected. Convinced of the need of an accident hospital in the district of miners and artisans, she did not rest until provision was secured, and there is now excellent accommodation in the institution whose affairs she will assist to administer.

### A COURSE IN HOSPITAL ECONOMICS FOR NURSES.

Mrs. Robb courteously sends us the syllabus of a course of study to be pursued at the Teachers' College in connection with the Columbia University, New York City, by a class of special students in hospital economics. The course has for its object the preparation of trained nurses to fill posts as teachers in training schools and as superintendents of hospitals. If pursued in addition to practical work no doubt such courses may be of some value; but neither in teaching nor in the management of an institution does theory go for much unless it is coupled with a sound practical training in the work which is taken up.

### IS THE NURSING PROFESSION OVERCROWDED?

According to the *Gentlewoman* the nursing profession is overstocked, there are hundreds of nurses in London who have nothing to do, and a nursing home in Paris is suggested as an opening for some of those who are unsuccessful in England. We are aware that the profession is overstocked, but not with good nurses. Of these the supply is still less than the demand, and seems likely to remain so. A nurse thoroughly trained in a good hospital need never be long out of employment. The cause of the overcrowding is that there is a plethora of half-trained nurses, and those who have received their knowledge from small and unrecognised training schools. A matron of a large and important hospital recently remarked, "Whatever becomes of all the nurses trained in small hospitals I cannot think." Of course, the small hospitals must have probationers, but they should either be drawn from a different class to those who enter a recognised school; or else should be girls too young to enter a large hospital and who wish to fill up their time with some preliminary training. Any girl or woman who really means to make nursing her life-work should carefully consider the choice of her training school, and shun small institutions unless she intends only to begin in them. "A three years' training in a general hospital which is a recognised training school for nurses, and which has a resident physician and surgeon," &c., &c., is a *sine quâ non* for any substantial success in the nursing world. If this is required in workhouses it surely will become the minimum standard for all nursing work. Of course, there are exceptions to this, as to all rules. For instance, with the L.O.S. certificate, and a year's training in a good hospital, a nurse may do very well indeed, and for district work a three years' certificate is not always necessary. But no nurse will ever obtain a hospital appointment worth holding with less. Many private nurses are unemployed because they are not thoroughly trained. Not long ago a family advertised, rather vaguely, for a nurse to attend to a chronic case in their home, at a salary of £1 1s. per week. They were inundated with replies, but there was not one from a first-class nurse.

### MORE NURSES NEEDED AT GRANTHAM WORKHOUSE.

At an inquest held the other day at Grantham Workhouse on an inmate of the infirmary, whose death was due to suffocation caused by a piece of meat lodging in his throat, it came out in the course of the evidence that while there are 63 beds and 60 patients in the infirmary, there are only two nurses. Each of these has, necessarily, to perform both day and night duty. When the jury were informed of the nursing arrangements they stigmatised them as shameful, and added a rider urging upon the guardians the need of at once engaging additional nurses. The rider does credit to the jury, but it is not at all creditable to the Grantham Guardians that they should have imposed upon the nurses of the infirmary a burden much greater than they ought to be expected to bear. Moreover, with such an inadequate staff the patients must obviously suffer from want of proper attention.

### NURSING AT TORQUAY.

The financial outcome of the successful garden party and fête held in the Princess Gardens, Torquay, on behalf of the Nurses' Institution and Rosehill Cottage Hospital is very satisfactory. The Nurses' Institution benefits to the extent of £100, and the hospital to that of £50. It appears that in Torquay, as elsewhere, it is often found that class distinction is a barrier to charitable effort and philanthropic aim. But in this instance there has been no such impediment. In the work of nursing, class distinction is happily unknown, and hence the combined effort at Torquay which has resulted in such a handsome addition to the funds of the Nurses' Institution.

### THE PARISH NURSE AT ARUNDEL.

Although there is a sharp division at Arundel between Anglicans and Romanists, the Rev. Walter Crick, the vicar, states that the Duke of Norfolk contributes a portion of the stipend for the parish nurse. The bulk of it is raised by the Anglicans, and the nurse acts directly under the vicar, assisted by a committee of ladies. It was at the instance of Mr. Crick, soon after his appointment, that the parish nurse was introduced, and her services have been warmly appreciated in the town.

### A JUBILEE NURSE FOR THURSO.

In all parts of the United Kingdom there is an increasing demand for the services of qualified nurses. At Thurso, it has just been decided by the leading inhabitants, with the Provost at their head, to engage a Jubilee nurse. There is no doubt that the experience of Wick has had an influence upon the people of Thurso. In the former town two nurses are employed at a salary of between £80 and £90 a year each, and they have proved such a blessing to the poor that their fame has been noised about in the district. A nursing association will, therefore, shortly be formed at Thurso, with the energetic personal support of a number of ladies, and promises of financial assistance from Sir J. G. T. Sinclair, the Parish Council, and other local bodies.

### SHORT ITEMS.

A nursing association has now been in existence for eight years at Aberlour, and the financial support is well maintained. At the annual meeting the other day warm testimony was given to the services of Nurse Jenkins.—The first annual meeting of the Raunds Nursing Association has resulted in the engagement of Nurse Duffield for another year. In her first year she attended 118 patients, and was on duty 1,566½ hours.—Two nurses, Miss Kate Lewis and Miss M. Frenhares, who volunteered for nursing small-pox patients in a San Francisco hospital, have died from the disease.

### Gynæcological Nursing.

By G. A. HAWKINS-AMBLER, F.R.C.S., Surgeon to the Samaritan Free Hospital for Women; Assistant Surgeon to the Stanley Hospital, Liverpool.

(*Continued from page* 308.)

THE AFTER-CARE OF CASES OF ABDOMINAL SECTION.

WHEN your patient has been put back to bed after an abdominal section, you take and record her temperature. Hot bottles are kept to her feet, under the arms, and alongside her to promote reaction. A watch is kept for chloroform sickness, and the strain lessened by supporting the abdomen gently but firmly during vomiting. The room is kept darkened and very quiet. If a drainage tube has been inserted, you will be shown how to suck it clear with a glass syringe, or preferably Tait's sucker. Usually, the sucker tube is dropped gently to the bottom of the drainage tube, and the blood and serum removed every fifteen to thirty minutes, the object being to keep the tube dry and assist in stopping hæmorrhage. After aspirating the tube, you place the sponge or pad over it and keep it gently held in place by pinning the many-tailed flannel bandage over it. The sucker is washed thoroughly, and then replaced in a solution of corrosive sublimate, 1 in 1,000 of water. In a similar solution you also keep a glass catheter, so that you can empty the bladder every six hours, or as often as directed. A book or chart will be provided for you, and in this you enter any observation you have to make, together with the exact hour at which it was made. Every two or four hours the temperature is to be taken and entered in the book and on a chart. The urine when passed or drawn is to be measured, and if peculiar must be saved in a clean, covered glass vessel for the doctor to see on his next visit. The frequency of respiration is often noted. Pain, if present, is described, and if severe is particularly mentioned to the doctor, who may or may not use something to ease it. As a rule we do not give our patients morphia after the dose some get before they leave the operation table. Unless you have instructions to the contrary, the patient is to be kept absolutely without food or drink. The thirst is sometimes cruel, but for the first twenty-four hours it is not usual to give more than a teaspoonful of hot water by the mouth, and perhaps only the lips are to be wetted or the mouth washed out. You may be ordered to administer an enema of water or salt water for the relief of the distressing thirst. If so, give it very slowly and without disturbing the patient.

Pain is sometimes very severe—less so after the vaginal operations that are now replacing section through the anterior abdominal walls with many surgeons. Occasionally flatulence is troublesome. In this case, and before it has become troublesome, you pass into the patient's rectum a glass rectum tube. If you have not one of these, use the vaginal tube of your enema syringe. Grease the instrument and press it gently against the anus. Pushing in a backward direction, it will readily enter, and may be left there by the hour together. It carries off wind and relieves considerably.

The important symptoms to watch for are hæmorrhage and inflammation—peritonitis. The former is indicated if the patient grows very restless, pale, with a quick pulse. A frequent glance at the dressings, which can be done without chilling or disturbing the patient, often gives you warning of such an occurrence in good time to send for help. Any sudden faintness or pallor, increasing weakness, restlessness, a temperature falling below normal, an increasingly rapid pulse, and sighing respirations, should be reported to the surgeon in attendance at once. Usually hæmorrhage is out of sight, and we have to depend on such indications as I have mentioned for its detection. Whether the symptoms be grave or apparently trifling, everything must be set down at the time it happens; do not defer a moment or shirk the most trifling note, which may have a serious meaning for the surgeon. So he will see on his visits the earliest signs of things to be dreaded and prepare for them. Another symptom for which you must watch assiduously is that of distension of the abdomen. The dressings cover the abdomen pretty extensively, but you can always notice the little gap below the sternum or breast-bone, the pit of the stomach. If this be depressed, as in the healthy subject, all is pretty well as far as distension goes; so with the flanks. But if the hollow disappear, and become drumlike and tense, serious mischief is brewing and must be taken in hand immediately. My own nurses have definite instructions, on the appearance of such symptoms, to give the patient an enema of turpentine, half an ounce in half a pint of hot soap and water. If this fails, she goes on to give the patient a Seidlitz powder or a teaspoonful of Epsom salts in hot water or mixed with a Seidlitz powder in aired water. This is repeated every two hours till the bowels act, or until she can communicate with me. It will be your duty to ascertain from the surgeon for whom you are nursing exactly what you must do in this respect, and when and how far you are to interfere. The above treatment is common to myself and many other surgeons, but it does not follow that the surgeon under whose particular orders you happen to be may approve of it, and as he is responsible to the patient, so you are responsible to him alone. Suggestions offered here are offered only in default of more precise instructions from your immediate superior, and cannot be allowed to supersede his wishes in the treatment of his own patients. Whatever preconceived ideas a nurse possesses must be entirely, willingly, and cheerfully subjugated to the will of the only man who knows exactly what he has to treat and has planned how he will treat it. There is no doubt that wearing the rectum tube gives great relief and prevents a good deal more serious results than mere pain. The turpentine enema given early by a watchful nurse, who soon learns when it is called for, is also of immense value; and these are cases where the surgeon, assiduous as his attendance may be, necessarily relies on his nurse for treatment in the emergencies that arise and that have to be promptly dealt with.

The rule as to food must be strictly adhered to. Washing the mouth is found comforting, sponging the face and hands, sniffing at a vinegar cloth, rearranging the pressure of the clothes, attention to the bladder and the rectum tube, the restful quiet of a sick-room, undisturbed by a noisy nurse who rattles fire-irons and mends the fire with the rowdy enthusiasm of the stoker of an Atlantic liner, or flares the gas up for the most unnecessary duty, who leaves her patient's hair unplaited, to tease her by getting into her eyes and ears, and a thousand irritating displacements—all these things make or mar the comfort of the sick room and tell against a patient who has often too much already lying between her and safety.

The slightest sign of hæmorrhage must be watched for and immediately acted upon. To raise the foot of the bed, keep the room cool, and the patient perfectly quiet are the first lessons of the careful nurse. To keep the drainage tube dry, to protect it from the admission of germs from an infected sucker, or through a bad cover, to empty it clumsily, all make for mischief. The movement of the patient calls for caution and extreme gentleness. Such services as are to be rendered, such necessary lifting as must be done, is done with great care and firmness.

Where she can be lifed in the sheet so much the better.

Where a small pillow under the hips or sides can ease her it is a little thing to try. And when convalescence comes care must again be exercised to prevent undue exertion till it be possible for the patient to undergo it. I have known a patient insist on climbing into bed unassisted the first time she sat up, and die within half an hour in consequence. You will be guided by your instructions, your knowledge, experience, and common sense, and not by the whims of a patient. I have heard of a lady who took the opportunity of the nurse leaving the room to get into a cold bath the morning after an ovariotomy. Constant care, watchfulness, tact are called for in convalescence, as even then we may have serious internal hæmorrhage as the result of undue exertion. The patient's belt, too, must be put on before she rises, and only removed, to change for a lighter one, after she has returned to bed.

### The Born Nurse.

### A STUDY FROM LIFE.

SHE is a feature in the private acquaintance of almost everyone. Usually she is healthy herself, and has seen little of sickness in her own family, beyond the inevitable influenza and an occasional attack of chicken-pox or mumps. This is often a cause of outspoken regret to the born nurse, who feels herself to have that within her which the small exigencies of a headachy day in bed, or a few feverish nights, cannot fully bring out. A "brain fever," now, or a "leg cut off," might show what is in her, she opines; because, as she gives you to understand, Providence has gifted her with an unprecedented delicacy of touch in pulling the creases out of a sheet, and her shaking up of pillows actually drew tears of admiration from the village doctor when she went down to see the gardener's sick wife. Also she knows how to make seven different kinds of lemonade and barley-water, and she is quite sure she would not faint at the sight of blood. And if that is not nursing, she would like to know what is.

You, a stranger within her gates, are laid *hors de combat* one day by a bad bilious attack, and ask feebly to be allowed to keep your room; yearning, as the victim of such an annoyance does yearn, only for peace and quiet, and a glass of "saline." Then is the born nurse in her glory. She brings up your breakfast herself, rustling with importance, and swelling with pride in the fringed d'oyly, the silver cruet-stand, and the fancy china service, displayed on the tray. "I always believe in serving everything daintily for the Sick," she says, putting at least three capitals into the word; "it tempts them to eat." And she uncovers a dish of buttered eggs, and attempts to feed you with a spoon. "What! not going to eat anything? Oh, but I'm sure it is good for you; eggs are safe in any illness, and even if you're sickening for typhoid, it won't harm you. . . . Well, if you won't—but remember, I shall insist on your eating your lunch; I am always firm with the Sick—they don't know what is good for them. You can take your tea, at all events; I'm so sorry there is not an invalid cup in the house, but I got you papa's moustache cup instead; it's nearly as good."

She has gone—thank goodness! She has smoothed over your quilt, and beaten up your pillows (which were quite comfortable as they were; but the born nurse is, vulgarly speaking, a whale on pillows), she has made your headache much worse by brushing and combing your hair in a "firm" and hearty manner, and told the housemaid to "bring in her pail and duster and tidy up at once." You close your eyes, and endure the rattling and banging patiently. The born nurse, at least, has taken herself off for a while. But you know you are not yet done with her; you feel a conviction that all day long she will persist in trotting in and out to "nurse" you, with a deadly activity of her own; that she will ask you in a hissing whisper if you want to be read to, if you will have your Bible, if you will let her take your temperature just once again, if you would like orange squash, hot milk, kali water, strawberries, eau-de-cologne, a cup of Bovril, a piece of toast. Then she will draw down the blinds, smooth your forehead exasperatingly with five "firm" fingers, and, planting herself with an elaborate parade of silence in a squeaking basket chair, sit like a statue of Memnon for an hour or more, visibly gloating over her own patient endurance, and watching you until your nerves are a-twitter like a grove of chaffinches in spring. Under such circumstances, the only course is to get well, or make a pretence of doing so, with the utmost possible rapidity, and let the born nurse take herself off to harry the parish rheumatics as fast as she can, glorying in her "cure."

Sooner or later, it occurs to the born nurse that a hospital is the real field for her talents; that hospital uniform is becoming, that the patients call down blessings on the golden head of "Sister Gwendoline" in the intervals of their agonies, as she flits down the ward, and that the most good-looking and eligible doctor always proposes to you before you have got through your first six months. So she writes to a paper (fashion, by choice), and asks how she is to get into St. Simon and St. Jude's, which she has selected for her *début*. The editor tells her rather wearily (he has to answer the question so often) that she must write to the matron of the hospital for particulars, and she does.

The papers she receives give her rather a shock, but she consoles herself for the apparent strictness of life and precision of rule by reflecting that she is an exceptional person, and that exceptions will doubtless be made in her favour. So she fills in her forms, has her interview, is accepted rather dubiously, and becomes a probationer of St. Simon and St. Jude's.

It is six weeks before she comes home again, and she has learned a good deal in that time—all that there is to learn about nursing, indeed; although she would have liked to finish out the three years, as a matter of form, if the hospital had been a well-conducted one. But it was not. She was never given a chance of learning anything—in spite of the amount of knowledge she has collected. The Matron was jealous of her youth and good looks, and the Sister of her ward was a most under-bred and impertinent woman. Her talents were entirely thrown away; she was given the most absurd and horrible things to do, and was shockingly overworked. The patients were the nastiest set of creatures she has ever seen in her life, and the doctors gave themselves the airs of dukes. No one made anything of her wonderful nursing instinct; no one appreciated her; she was misunderstood from beginning to end, and she will never enter a hospital again.

For the rest of her life, all the same, the born nurse poses as "hospital trained," and patronises "untrained nursing" ineffably. She keeps her bonnet and cloak, and sometimes puts it on when she goes district visiting, to give herself an official appearance and show how well she looks in dark blue with a fluttering veil. She talks shop unweariedly in any and every company, her delight in things "bluggy" exceeding even that of the immortal Toddie. She laughs pityingly at her mother and aunts when they hold up their hands and chorus "Don't!" to her vivid description of somebody's intestines as they appeared when temporarily reposing in a bowl of warm water; and she twits her younger sisters with squeamishness when they refer to her word-picture of a gynæcological operation as "really too horrid." On the whole, she manages to be about as much of a discredit to the profession of nursing generally as one solitary young woman can. The fewer born nurses that the world of the sick numbers in future days, the better it will undoubtedly be for the born nurse's antithesis—the genuine nurse.

### French Hospitals.

By an Occasional Correspondent.

### THE PROBLEMS OF THE KITCHEN.

I do not think that in England enough importance is accorded to the kitchen. Since the well-being of patients depends really more upon food than upon medicine, or quite as much upon food as upon those bodily attentions which are included in the term "nursing," it is but common sense to honour the kitchen and to give some of that dignity to the kitchen staff which has been found to be so valuable in increasing the standard of work in the wards. Feeling strongly, therefore, that a "kitchen sister" should rank on a par with a "ward sister," and that her chief assistants should take similar status to that of ward nurses, I was in many respects charmed by the management of the kitchen at La Charité Hospital.* If it be true that the French nursing staff falls below the average nursing staff of a good English hospital, it is none the less true that the *personnel* of the kitchen is higher.

Going to the kitchen after leaving the wards I was most kindly received. The two "kitchen supers"—if I may call them so—one male, the other female, took the utmost pains to show me everything. The kitchen utensils were largely of copper, and were beautifully clean. That I might be quite sure that it was "liver" which I had seen served in the wards, I asked to inspect the larder, and found this very repulsive-looking food safely reposing amid lumps of ice. The larder also contained large bins similar to those in a corn merchant's shop, filled with dried foods. I noticed specially golden maize meal, vermicelli, peas, lentils, haricots, and flageolets, but I missed our old friend oatmeal, which under one or other of its many forms has become such a feature in the English dietary. I missed also the stores of dried fruits, such as raisins and currants and figs and dates and prunes, which we have in England now learned to look upon as an important part of our national food.

If I turn for a moment from the kitchen of La Charité at Paris to the kitchen at the hospital at Amiens, it may make this diet question a little clearer. When the surgeon who was taking me round the Amiens Hospital understood that I was interested in the kitchen, he introduced me to the sister who was in charge of it, and she, when she knew that I was English, clapped her hands and said, "Eh, but we have an English sister here; she will so like to see you and give you information." And straightway she went off to find her.

It was very curious. Sister Elizabeth had come to Amiens from London a dozen years before, and since then she had neither heard nor spoken her native tongue! As a nun belonging to a nursing order she had for all these years been working in this French hospital, and probably will continue there to the day of her death.

"Now, sister," I said; "do you mind telling me just how you feed your patients here?"

"Well," she replied; "in the first place we have an allowance of meat from the administrative bureau, which works out to 250 grammes per head."

"Before or after cooking?" I asked.

"Oh, before cooking; we reckon to allow a quarter for cooking and a quarter for bones and waste, so that the actual amount averages about 125 grammes of cooked meat per diem."

"What sort of meat?" I inquired, remembering the liver dinner at La Charité.

"Oh, beef," and then, after a little discussion with the kitchen sister, she added, "but we have every week so many sheep allotted to us by the town council, and we use the flesh of these sheep for any of the very sick patients who can't eat beef."

It struck me as a quaint picture for the municipal fathers to go down to their flocks and to pick out a few sheep and then to drive them down to the hospital as a sort of sacrificial offering at the temple of Æsculapius, and the fragile-faced nuns to carry in the bleeding limbs to the kitchen to make broth withal! What I suppose was really meant was that so many whole carcases were sent weekly to the hospital, and that out of these the sisters had to do their best to provide mutton broth and chops and such like.

"Now about the times of meals and the daily menu?"

"First meal 7 a.m.," she answered.

"Soup, I suppose, and bread?"

"Yes," said Sister Elizabeth, "or coffee or chocolate."

"Come now," I replied, "that is nicer than eternal soup; but is it alternate mornings, or how is the choice given?"

"There is not much choice I am afraid," she replied. "In the surgical wards it is always soup and bread, and in the medical wards it is soup or coffee or chocolate, according to the doctor's orders."

"And the next meal?"

"At 10 a.m. the second meal is served. It consists of soup, which may be a meat soup or a maigre or milk soup, like tapioca or vermicelli. This is followed by meat and vegetables and bread, and either beer or wine or milk is given to drink."

"Yes; and when are the patients fed next?"

"At 4.30 p.m., and they have then a repetition of the last meal."

"And supper?"

"There is no supper. There is nothing to eat in the ordinary way from 4.30 p.m. to 7 a.m. the next morning."

"But have you never any patients on one or two hour feeds?"

"Yes, sometimes, and then, of course, they are fed according to their needs; but the regular hospital dietary is what I have described. We give, however, a small feed of milk at eleven p.m. to a few patients who are exceptionally ill."

"Now, sister, you are English, and you will understand the wonder I felt at the absence of all puddings and pies and fruit and fresh vegetables in the Hospital of La Charité. Is it the same here, and if so, why is it?"

A dreamy, far-off look of the old days in England came over the sister's face as I talked of puddings and pies, and a gleam in her eyes told me that her life of ascetic devotion had not quite extinguished all the natural instinct for a dainty dish. It was but for a moment, and then came the same measured, quiet tones—

"It is the custom of the country. Here the doctors think that fruit is not good for health, and I do not think that it is much eaten anywhere in France after mid-day."

"And why don't you use vegetables?" I continued. "I always had the idea that France was the land of beautiful vegetables, daintily cooked, and delightfully served in a multitude of tasty ways."

"That may be true as to just a few fresh vegetables; but the custom of the country is to use dry vegetables, not fresh ones—haricots and peas and lentils and root vegetables, and such like are our stock supply; but if you come into our kitchen for a month you would never see the pile of cabbages or sprouts or cauliflowers that are to be found in all English kitchens. No, I do not know why, but it is so. It is the custom of the country."

I fancy that English cooks would be glad to be rid of the mass of work which is entailed with preparing fresh vegetables, and the heaps of refuse and rubbish which are brought with them; but I think the patients would suffer, and the general health of our community would deteriorate, if we gave up the universal use of the wholesome greens with their wealth of soluble salts. This lack of fresh fruit and vegetables may be one reason for the French suffering so much from "nerves."

* The Hospital de la Charité has 516 beds.

### Echoes from the Outside World.

### AN OPEN LETTER TO A HOSPITAL NURSE.

Since the days of the Phœnix Park murders no news has caused such horror and indignation as the verdict in the case of Captain Dreyfus. When first the fact that "a body of military scoundrels," as someone has called them, had dared to pronounce the prisoner guilty, and that he had been condemned to ten years' imprisonment, was made public, there was only one sentiment expressed—that of angry surprise. For whatever the known desire of some of the judges of the Rennes Court Martial may have been, it was not anticipated that they would dare to commit such an outrage against justice. A Sunday I felt as if I had heard bad news of a personal friend, and when I went into St. Paul's Cathedral in the afternoon I could at once see by the grave looks and the expression of tension on many faces that the fate of Dreyfus was the topic uppermost in the minds of most of the worshippers. Canon Scott Holland was listened to with rapt attention, not only because of his powerful discourse on the divisions amongst the Israelites, but also because we all knew, in some subtle manner, that he, too, was thinking of a man far away in a French cell, weighed down by a bitter burden. When, at last, the allusion which we were all expecting came, a rustling sound, a perceptible stir, seemed to roll from one end of the vast edifice to the other. The men sat up straighter, as if bracing themselves for battle; the women bowed their heads to hide their rising tears. Though the language in which the preacher spoke of "France standing at the judgment bar of civilisation" was emphatic and decided in its condemnation, there was obviously a laudable desire not to stir up the passions of his hearers, or in any way to increase the general feeling of excitement. As he hinted at the possible clemency of the President of the French Republic, I could not help thinking how little such clemency, should it be exercised, would really meet the needs of the case. How is it possible to pardon anyone who has not committed a crime? In any case, to withdraw the punishment whilst still insinuating belief in his guilt would hardly be a kindness to a man who values, as Dreyfus does, for the sake of his wife and children, the honour of his good name.

Naturally, reprisals are suggested, but to punish individuals for the crime of the nation is most unfair. Nothing could be more illogical, or reprehensible, than the behaviour of the contractor in Chicago who, on learning the verdict in the Dreyfus case, at once discharged all his French employés. Yet, unless matters assume a very different aspect before 1900, the Paris Exhibition is likely to be boycotted by us, not because England feels vindictive, but because it would give English men and women no pleasure to seek enjoyment in a capital which rejoices at injustice done to an innocent man, and because English exhibitors would only feel humiliated, not gratified, at receiving medals of distinction from those whom they had ceased to respect. Austria seems to have decided not to take any part in the forthcoming show, but it is to be hoped that Great Britain will not lightly pursue such an extreme course. Germany, in spite of great provocation, has declined to resent the affront to the Emperor, by having anything to do with the boycott.

The Barking School Board, in building themselves a fine new school, have set aside a small building for the special teaching of deaf mutes. At present there are only four little scholars, which is probably not because that part of London is free from children whose lives are thus clouded by misfortune, but because the possibility of their being able to get instruction suited to their particular infirmity is not yet generally known, and the Board is anxious that the existence of such a school should be more widely noised about. District nurses very soon learn to know not only the complaints of those whom they can help by their ministrations, but also all who are in any ways afflicted, incurably or not. So I am mentioning the fact of the recent development at Barking, not only because it is always pleasant to note how the sick in mind or body are cared for, but also because nurses working anywhere in the locality may be able, by telling of the school, to benefit some who at present are plunged in the awful darkness of complete isolation.

I believe experience has proved that boarding workheuse children out in different cottages with a woman called a "mother" to look after them is the most satisfactory plan, and secures for the children, on the whole, a better upbringing and a happier existence. But the evidence lately given before the Bridgend Guardians shows that there are "mothers" and "mothers," and that the fact of a woman being designated by the title which should mean to a child all that is sweetest on earth, does not necessarily secure the corresponding virtues. It appears that one of the "mothers" in her love of cleanliness had determined to have the child under her care free from dirt, for once, so she scrubbed the child's face with a scrubbing brush, and thus removed the whole of the skin! Such inhuman conduct is enough to make that child rejoice in dirt to the end of its days, since in its nfantile mind cleanliness and cruelty must seem synonymous.

Have you seen the new earrings? I do not ask if you wear them, because except "off duty" you would not be likely to do so. Gipsy earrings and a nurse's costume would be most incongruous, and I should not advise anyone except those who possess the right type of face to adopt the big golden circlets which are now being worn in the ears by a few women, notably Miss Vanbrugh, the actress. They are strongly suggestive of fancy dress, and it is quite surprising to the onlooker who first notices these appendages to find that the wearer is not masquerading, either as a gipsy or as a Creole maiden. For evening wear the effect is pretty, as the gentle swing of the golden rings seems to harmonise quite sufficiently with the low neck and unusual style of attire, but if worn with a morning blouse and collar and cuffs the appearance is distinctly ridiculous. The jewellers, who are thus making one more effort to introduce earrings as a fashionable article of attire, are hopeful that those who have had their ears pierced will be so glad to show off the many valuable precious stones at present lying idle in their jewel caskets that they will at once jump at the idea, and, by themselves adopting it, soon induce others to do likewise. But I think they forget the hundreds of women who, if very young, have never had a hole bored through their flesh at all, and, moreover, do not want to.

The final issue of the now historic Cabinet Council is, as I write, still in suspense, and many contradictory reports about the different commands are current. A rumour that Sir George White is to have the command of the Indian contingent for Natal, for instance, lacks authority, though it may be only premature. No one, we can all imagine, is better fitted for the position than the late Commander-in-Chief in India, and the significance of his appointment would not be lost sight of by the shrewd Boers. In Durban the choice would, I know, be received as glad tidings, and General Simons would not in the least resent it as implying his supersession. President Kruger is not the kind of man to allow meetings of cliques got up by nobodies, whether in London or Manchester, "to protest against war," to blind him as to the real sentiments of the British nation, of which the unanimity of the Cabinet is only one of many signs. If, as I always think, manifestations of Stock Exchange feeling are not particularly valuable, the patriotic speeches of several well-known Liberals indicate, without doubt, that the Opposition and the Government are, as they should be on this question, of one mind.

### Across the Seas.

### AN ANTIPODEAN HOLIDAY.

By the Matron of an Australian Hospital.

After a year and eight months of hard work in a large hospital, nature begins to cry aloud for a respite, and by unmistakable signs to indicate her intention of having one, or else taking her revenge in her own way. Wisely (for nature had previously taught me to respect her timely warnings), I set about gratifying her and pleasing myself.

Now, our Australian continent does not lend itself readily to a lazy or tired tourist. What beauty spots we have need finding out, for few are easily accessible as yet—excepting, perhaps, Sydney's beautiful harbour and one or two resorts near our other cities. Tasmania I had "done" thoroughly, Japan, I regretfully decided, was too far away, the Pacific Islands not desirable just then, but New Zealand, the land of natural wonders, lay only 1,200 miles away. After looking at the pros and cons, I decided to go there, especially as my most trusted friend assured me, by specious arguments, that four and a half days by water was of slight account, mal-de-mer being only a matter of will. I am, however, now prepared to assert that mal-de-mer is not a matter of will at all, that it is in no way related, directly or indirectly, to personal will power, but is controlled by laws of gravitation, over which the will is powerless. For three hours after I embarked I sat and sternly called on my will to do its duty, which it did until those same laws of gravitation proved the stronger and laid me low for three sorrowful days.

The Pacific Ocean is the gravest misnomer I have come across; its name amounts to positive deceit and cunning. Our early navigator must have had a fertile imagination and a grim sense of humour when he so called it. I can only suppose he meant it as sarcasm.

However, I reached Wellington one charming afternoon when the winds were happily at rest and the lights and shadows chased each other over the beautiful hills by which the city is surrounded. I had left a drought-stricken continent, but here I found green grass waving high, happy rills rushing down the mountain sides, cool, mossy nooks, where a wealth of fern made a rest to the eye and a delight to the spirit, where the forget-me-not and primrose grew without care or watering, and where, best of all, the kindest of friends united in making my stay a store of happy experiences.

The houses are all built of wood, and look to European eyes very fragile and unsafe; but here, where earthquakes are common, they are proved to be the safest of structures. The Government House, Government buildings, churches, theatres are also all of wood, and some very picturesque and pretty. The seat of government is in Wellington, and it is a busy, bustling little city. After climbing the highest mountain and seeing its view, I began to arrange for further afield. Then followed in quick succession, as day succeeded day, many and varied beauties—lakes, rivers, waterfalls, gorges, mountains, plains, geysers, volcanoes, and cataracts.

I found the hot lakes region a fairyland of wonder and interest. Here, side by side, lie pools which are icy cold and others hotter than boiling point. Every colour of the rainbow is reproduced in liquid and solid form; fairy geysers toss their feathery plumes high into the sunny sky, and low mutterings and ominous detonations speak of unknown and sinister demonstrations in the future.

Picturesque Maoris squat at the door of their whares, or sit placidly in the warm pools. It is truly a lotus-eaters' existence which these dusky natives live, subsisting chiefly on the bounty of tourists, and regarding work as a painful incident—fit only for Europeans.

Travelling is very expensive and very slow in New Zealand, and one must be content to resort to any means of transit, railway, boat, buggy, canoe, or horseback.

### A Reminiscence of Florence Nightingale.

In the shadow of Mount Egmont I came across an old Crimean veteran, whom I discovered to my great delight had been a contemporary of Florence Nightingale's at Scutari. Many and interesting were the incidents he narrated of the hospitals, the war, the officers, and soldiers. I learnt more about hospital work in the Crimea that one evening than all my reading had ever taught me. My friend had been male hospital superintendent, whilst Miss Nightingale was female superintendent, and through him I learnt to know the living, breathing, winsome woman, where before I had simply gazed afar off, through the mists of time, at a heroine who had done noble deeds. I realised, as never before, what England lost during these few fearful weeks when want and disease carried off her bravest sons, and sorrows reigned which the few devoted workers like Florence Nightingale and my old friend could do nothing to relieve. One heard the heart of England throb at its loudest when the ex-superintendent said: "And then supplies came. All England poured their offerings out at Scutari; miles of provisions, medical comforts, clothing, lined the hospital. England could not do enough to retrieve somebody's mistake, could not sufficiently show her devotion for her heroes. There was a plethora where before had been amine, and our men were soon to the front again."

I visited every hospital I came across in New Zealand, and found most of them well up in modern nursing and treatment, and in almost every instance they occupy splendid sites, especially that of Auckland, where the hospital is built on an eminence commanding a view of the lovely harbour and surrounding country, including many little islands dotted about near and far. Close to this hospital is the nurses' home and a handsome new building to be devoted to children and convalescents.

Then at last I returned to my own dry, dusty city with a feeling of pity for those less fortunate than myself who had not enjoyed a break in the summer's heat and drought. But the pity was mingled with pride as I looked around me when I got back to work again, and felt that I had seen few to equal, and none to surpass, that spacious and beautiful building which lies under my own special charge.

### Novelties for Nurses.

### "THE SILENT" PATENT ATTACHMENT FOR THE NURSE'S CHATELAINE.

(Messrs. Maw, Son, and Thompson, Aldersgate Street, E.C.)

This silent attachment is not an undemonstrative love affair, as its name seems to imply, but is a useful little silver-plated case in which are contained the appliances required in making the clinical observations which every nurse has to record. It contains a clinical thermometer, a pulse glass (which can be seen without removing it from the case, where, however, it is all the time secure from injury), and a pencil. The case is neat and light, and is likely to prove useful. Why it is called "silent" we do not know, unless it be that if the instruments it contains were all to be kept in separate cases dangling from the chatelaine much rattle and jangle might be produced, all the annoyance of which is saved by using the "silent" attachment. Tho price is 6s., including the thermometer. We do not much like a nurse to use pulse glasses which run only for a quarter of minute. The time is too short for accuracy in counting the pulse, and quite too short in estimating the respiration. Half a minute is much better, and even a minute is none too long.

### Resignation.

Hertford and Ware Joint Hospital.—Miss McIntyre, matron, resigned on the 5th instant. She has opened a private home in Hastings.

### Nurses at Tracheotomy Drill.

The diphtheria nurses at the South-Eastern Fever Hospital have lately had a very busy time in perfecting a tracheotomy drill on the principle of a fire drill.

"The idea commenced," writes one of the nurses, "in this way. We had a holiday locum who scoffed openly when told that after receiving an emergency case in a ward for immediate tracheotomy all due preparations for the doctor's knife could be made in the space of two and a-half minutes. Before he left us he had the opportunity of seeing the preparations carried out, and operation completed, with patient breathing through the tube, in three minutes. This suggested to Dr. G. the idea of a competition, and he took the drill in hand with the assistance of his charge nurses, who do not themselves take practical part in it. When it is perfected a medical superintendent from some other fever hospital will act as examiner, and marks will be given for the most efficient as well as quickest preparation. Of course everything needful is kept in each ward in the handiest possible form. To each nurse is assigned a distinct portion of work, as the preparation of the patient, the placing of table screens in the strongest available light, or the arranging of instruments, &c. At first various little hitches occurred owing to nervousness and flurry, but after a little practice the thing was done methodically and quickly without rush. The order "preparations for tracheotomy!" is sometimes issued unexpectedly in the midst of ordinary routine, and usually meets with a capable response.

"As the life of a patient often hangs on a speedy operation the importance of this drill is not difficult to demonstrate. Demonstrations have been given in the lecture-room as well as clinically on emergency treatment, and the proper manner of holding a patient for the operation when only one surgeon is present, as not unusually occurs in private nursing. The boy patient who has been the model for the lecture-room demonstrations was a perfect little brick. Bribed with pence beforehand, he did not flinch when he saw the scalpel descending perilously near, and even the nurses present trembling lest the doctor, in his enthusiasm for the subject, should forget that the case was not one for genuine tracheotomy. When questioned later concerning his lecture-room experiences, he said, 'I saw millions of nurses there, and didn't the doctor give them wiggins! He says they don't know how to 'old us proper and didn't orter 'old us like they do.' He was discovered later on a garden seat practising the imaginary operation on a fellow patient with a broken tin whistle. The practises have been a huge success, and all the diphtheria nurses intend to come out first in the exam."

### Minor Appointments.

St. Leonard Infirmary, Shoreditch.—Miss Jane Green has been appointed Ward Sister. She was trained at the Poplar and Stepney Asylum, and subsequently held appointments as assistant nurse at Hackney, and charge nurse at the Park Hospital.—Miss Elizabeth Wilkinson has been appointed Staff Nurse. She was trained at the Sheffield Royal Infirmary, and has since been attached to a private nursing home at Sheffield.

Alcester Union Workhouse Infirmary.—Miss Margaret Gouldson has been appointed Superintendent Nurse. She was trained at the General Infirmary, Chester, and has since been charge nurse at Bromley and Beckenham Joint Hospital.

Dewsbury General Infirmary.—Miss M. M. Greene has been appointed Charge Nurse. She was trained at the General Hospital, King's Lynn, and has since been charge nurse at Croydon, and home sister at Victoria House, Gillingham Street, London.

Fulham Infirmary.—Miss Florence M. Moles has been appointed Sister. She was trained at Guy's Hospital, and has since been charge nurse at Park Hospital, Lewisham.

### For Reading to the Sick.

"The peace of God, which passeth all understanding, shall keep your hearts and minds through Jesus Christ."—*Phil.* iv. 7.

"His will is our peace."—*Dante.*

We ask for Peace, O Lord !
Thy children ask Thy Peace !
Not what the world calls rest—
That toil and care should cease,
That through bright sunny hours
Calm life should fleet away,
And tranquil night should fade in smiling day ;
It is not for such Peace that we would pray !

We ask Thy Peace, O Lord !
Through storm and fear and strife,
To light and guide us on
Through a long, struggling life ;
While no success or gain
Shall cheer the desperate fight,
Or nerve what the world calls our wasted might,
Yet pressing through the darkness to the light.

It is Thine Own, O Lord !
Who toil while others sleep,
Who sow with loving care
What other hands shall reap.
They lean on Thee entranced
In calm and perfect rest.
Give us that Peace, O Lord, divine and blest,
Thou keepest for those hearts who love Thee best !

*A. Procter.*

What is sickness but God's own method of calling the soul to "come apart" with Him as unto a desert place, and to "rest awhile"? In the midst of health and strength, is it not so that there are many "coming and going," and there is little leisure to feed on that "Word of God" which alone can stay the soul.—*Butler.*

I desire the joy of peace, the peace of Thy children I crave, whom Thou feedest in the Light of Thy consolation.

O righteous Father, and ever to be praised, the hour has come when Thy servant is to be tried.

O beloved Father, it is meet that in this hour Thy servant should suffer something for Thy sake.

O Father, always to be adored, the hour is come, which from eternity Thou didst foresee should arrive, when for a little while Thy servant in His outer life should be oppressed, that in his inner life he might ever live before Thee ; that he should be for a little while slighted, humbled, and in the sight of man fail, and be broken with sufferings and infirmities so that He may rise with Thee at the dawn of the new Light, and be glorified in Heaven.

O Holy Father, Thou hast so ordained it, Thou hast so willed it, and it has come to pass as Thou didst ordain.

Grant that I may die to all things which are on the earth, and for Thy sake love to be despised, and to be unknown in the world.

Grant to me—above all things to be desired—that I may rest in Thee, and that my heart may find its peace in Thee.

Thou art the peace of my heart, Thou its sole repose ; out of Thee all things are hard and unquiet.

In this very peace, that is, in Thyself, the Sole, the Supreme, the Eternal Good, I will sleep and take my rest.

—*Thos. à Kempis.*

### To Nurses.

In order to increase and vary the interest in the *Mirror*, we invite contributions from any of our readers in the form of either an article, a paragraph, or information, and will pay a minimum of 5s. for each contribution. All rejected manuscripts are returned in due course, and all payments for manuscripts used are made at the beginning of each quarter, *i.e.*, January 1st, April 1st, July 1st, and October 1st.

### Everybody's Opinion.

[Correspondence on all subjects is invited, but we cannot in any way be responsible for the opinions expressed by our correspondents. No communication can be entertained if the name and address of the correspondent is not given, as a guarantee of good faith but not necessarily for publication, or unless one side of the paper only is written on.]

### THE SALARIES OF NURSES IN THE TROPICS.

A CORRESPONDENT, who ought to know, writing from Hong Kong under date July 21st, in reference to the salaries of nurses in the tropics, says that the nurses in that colony receive during their first year £48, and the head sister, or matron, £72. This statement our correspondent makes in consequence of a note in these pages on June 3rd, founded on information supplied by the Colonial Nursing Association.

"ANIN," writing on the same subject, says: Your information with regard to the private nurse is correct, but it is also stated "that nurses holding a Government appointment receive a salary varying from £100 to £250, with the usual advantages accorded to all Civil servants." Were it not for the latter clause, the £100 might be assumed to represent the relative value of the post, but it is only natural that the general reader, without explanation, should infer that the commencing salary is £100, plus "the usual advantages." I know for a fact that in the large Government civil hospital at Hong Kong there are nine sisters and a matron all receiving less than that sum. The latter, having completed her fifth year of service, is in receipt of 85 dols. per mensem (including compensation) plus the usual advantages; while juniors start at 40 dols. per mensem, plus compensation, about 10 dols., and their salaries rise by small annual increments until the maximum sum (about £80 English) is reached in three years. Compensation is a local allowance upon the fluctuating dollar, and its continuance is entirely dependent upon legislative vote. Recently the rates of living in this growing colony have increased so enormously that the majority of Civil servants have found their salaries totally inadequate to meet their expenses, and a petition for a higher scale has been forwarded to the governing body. As at least seven members of the present community obtained appointment through the Colonial Nursing Association, from whose hon. secretary your statistics were drawn, the inference is that the net value of the post is comprehended in the £100, or that there has been some mistake in the figures quoted.

### NURSES AS STEWARDESSES.

"A. C." writes: I see in a recent issue a query respecting the post of stewardess, and as I sailed once in that capacity may I give a word of warning such as I should have been glad of? Two years ago, I, being a certificated nurse desirous of a change and longing to travel, applied at the office of one of the great shipping companies, and satisfactorily filled in the form supplied. Three weeks after I sailed, but before reaching Gravesend, I discovered my mistake, missing the respect to which as a nurse I had been accustomed. The life while afloat is not exactly what a self-respecting person would like. The heat below is stifling, but the stewardess is not allowed on deck among the passengers, even though conspicuous by uniform. There is no place to take meals except your cabin, which is usually wedged in the centre of the ship without porthole or any communication with fresh air, and ours was infested with rats, which spoiled two pairs of my shoes by gnawing. But the early experience was the better, and far preferable to that later on when the ship was at anchor, often for a period of three weeks. My experiences then I could hardly describe here, but would be pleased to do so to anyone who wishes to know what to expect. I was away three months, and was glad to return to nursing.

### BREAKFAST IN THE BEDROOM.

"BEE" writes: I should like to add a few words with regard to "Edith's" opinion of "Breakfast in the Bed-room," and being a private nurse of three years' standing I can speak from experience. During that time I have, on the whole, always received that kindness and consideration which is due to a nurse who does her work conscientiously, and not once during that time have I been asked to take breakfast in the bed-room. Should this occur I should consider it my duty to flatly refuse to do so, if only from a sanitary point of view. It is a nurse's duty to make use of her training and thus kindly teach the ignorant what is really essential as regards taking food in a wholesome atmosphere. Is not a nurse's health of importance? Is it not a rule of all, or most, good institutions that a nurse may not take her meals in the patients' room or with the servants? Surely there are very few cases where some member of the household cannot relieve the nurse for her meals, when she will return to the sick room brighter and more cheerful for the little change of atmosphere and surroundings, and it should always be a pleasure for the friends to have a short time alone with the sick member of the family instead of saying, "Oh, nurse is there." With regard to meals in the kitchen, if the nurse is used to that class of company, of course she would find it difficult to free herself from the gossip she would meet with there; but I am sorry for the poor patient who must put up with this class of nurse. If a nurse is considered fit to nurse a sick member of a family, who requires at all times such tender care, surely she is fit to mix with and take her meals with other members of the family, who, as a rule, are less sensitive than the sick and suffering.

"EDITH," replying finally to "Surprised," writes: Perhaps "Surprised" is so lucky as to belong to one of those institutions who arrange for their nurses to be treated as "One of the Family." I believe there are such. The one I was with five years did not do so, although it was one of the oldest in London. But perhaps things have changed since those days—nine years since or more. I know I used to dread each new case, and go in fear and trembling as to how I should be received. Possibly, if I had possessed what a celebrated doctor told me I lacked, "bounce and assurance," I should have got on better. "Surprised" asks, How can I make arrangements beforehand? Do I interview the lady of the house? When I get cases in the neighbourhood of my own home there is no doubt on the subject; no one knowing my family would ask me to nurse in their family on any other terms. Such persons have generally called and asked if I would come to them, or written and asked me to call on them first. One such case I had and held for four years at two guineas per week for the whole time. In this instance I was certainly regarded as a lady on terms of equality with the family. I am now nursing a sister of a bishop of the Church of England, in whose house I am treated as an equal, and am very happy. Where I do not know the people beforehand (as in this case, obtained by advertisement) I state when writing in answer my terms, references, and all particulars, and send photo, asking questions in return as to their family arrangements and what is required from myself; and, if in any doubts, ask for references from them—shall I say as to their characters? Of course, from those who do not mean to deal fair with you, you hear nothing further. Those who mean to treat you fairly are as frank with you as you have been with them, and like you all the better for being particular where you go. Of course, I do not, as many nurses do, confine myself strictly to only undertaking nurses' duties, but undertake any duty, as a daughter would or a sister, from arranging flowers to teaching in the Sunday-school, and making myself generally pleasant and obliging.

### Appointments.

NEW CONNAUGHT HOSPITAL, ALDERSHOT.—Miss Browne has been appointed Superintendent Sister. She has for some time been in the Army Nursing Service, her last post being that of Superintendent Sister at Herbert Hospital, Woolwich.

HERBERT HOSPITAL, WOOLWICH.—Miss Garrick has been appointed Superintendent Sister. She was trained at the London Hospital, and has since been an Army Nursing Sister,

BOROUGH FEVER HOSPITAL, BURTON-ON-TRENT.—Miss Henrietta Anderson has been appointed Matron. She was trained in the Burton Fever Hospital, and has since filled the position of probationer, assistant nurse, and charge nurse.

### Notes and Queries.

**The contents of the Editor's Letter-box have now reached such unwieldy proportions that it has become necessary to establish a hard and fast rule regarding Answers to Correspondents. In future, all questions requiring replies will continue to be answered in this column without any fee. If an answer is required by letter, a fee of half-a-crown must be enclosed with the note containing the enquiry. We are always pleased to help our numerous correspondents to the fullest extent, and we can trust them to sympathise in the overwhelming amount of writing which makes the new rules a necessity.**

**Every communication must be accompanied by the writer's name and address, otherwise it will receive no attention.**

*Workhouse Nursing.*

(203) Having read frequently in your paper, THE HOSPITAL, about workhouse infirmary nursing and raising the tone of same, may I ask to whom should I apply? To the County Council or the Board of Guardians?—*Flight.*

You have asked no question, but your letter suggests that you wish to apply for work in an English Poor-law Institution, with a view to raising their tone. The information you want is therefore as follows: Each poor law infirmary is under the control of the parish guardians, and the guardians are under the supervision of the Local Government Board, Whitehall, London. There are various associations desirous of raising the tone of nursing in these infirmaries by inducing capable, highly trained nurses to accept employment in them. But the conditions of work are often onerous and dull, so that the problem of obtaining and keeping good nurses in many workhouses is a vexed one. Vacancies are advertised as they occur, and a large number will be found weekly in our columns.

*Testimonials.*

(207) "K. B." would be very grateful if the editor would inform her whether a nurse, fully-trained, with a three years' certificate and excellent private references, and who is attached to a large nursing institute (70 nurses) under the superintendence of a fully trained matron willing to recommend her, would be ineligible for a post in a London hospital unless she also has a reference from the matron of the hospital where she trained. This is a matter of importance as the matron's of "K. B.'s" training school was noted for her harsh, often unfair, and therefore detrimental recommendations. "K. B." hears that hospital posts are not to be obtained by nurses from private institutions even if it is only a short time since they left a hospital, and that taking up private work for a year is an injury to a nurse's prospects.

It is usual to send all testimonials, especially such an important one as that from the matron of the candidate's training school, when applying for any position, but if the circumstances were explained the absence of the matron's certificate might not render "K. B." ineligible.

*Asylum Nurse.*

(2 8) Will you kindly tell me if you think I am suitable for asylum work? I am twenty years of age, and have been a governess. I have had anæmia, and also have a cataract in my left eye, which cannot be removed. I am able to do needlework, and can see as well as anyone. I am only five feet in height.—*E. W.*

We fear that many matrons will consider you unsuitable.

*Q.V.J.N.*

(209) Would you kindly tell me how I could become a Queen's Nurse, also what are the rules, or where I could get them? I have had twelve months' general training, nine months' fever, and four years' private nursing attached to a "home."—*Queen's Nurse.*

Write to the General Superintendent, Queen Victoria's Jubilee Institute for Nurses, Queen Katherine's Hospital, Regent's Park, N.W.

*District Nursing.*

(210) "Daisy" would like to know how she could obtain the name and address of a district nurse who would let her lodge with her and get a little insight into nursing the poor. She would be quite willing to pay nurse for her trouble.

A short advertisement would probably answer "Daisy's" purpose best.

*Pensions.*

(211) I want, when trained, to join some branch of nursing service to which a pension is attached. At that time I shall be too old for the army nursing service, so I should be glad of any information as regards the terms offered by workhouses, lunatic asylums, and fever hospitals in England.—*Pension,*

We cannot recommend you to rely entirely upon the Superannuation Act in the workhouses of Great Britain. Individual institutions in both the other branches you name make some arrangement with the Royal National Pension Fund for Nurses as regards pensions for their employés. As a nurse, however, you can become a direct member of the Fund yourself, without any restrictions as to your choice of nursing. Apply for particulars to the Secretary, 28, Finsbury Pavement, E.C.

*Deaf and Dumb.*

(212) Would you send information how to get a child into a deaf and dumb home for training, and if you have a book on prison nursing?—*F. M. Long.*

You will find a list of institutions for the instruction and care of the deaf and dumb in "Burdett's Hospitals and Charities" (5s., from the Scientific Press). Your parish guardians are entitled within certain limits to pay the expenses of a child of humble station. 2. There seems no book on the subject of prison nursing; but a series of articles upon "Prison Training and Administration" appeared in THE HOSPITAL, beginning March 12th, 1898, which dealt with the matter.

*Silent Shoes.*

(213) As I am flat-footed, and therefore cannot wear soft felt slippers without heels, could anybody kindly tell me where I could obtain those with heels or any other sort of silent shoe suitable for night wear in hospital wards?—*Flatfoot.*

Nearly all shoemakers catering for the custom of nurses have a selection of shoes suitable for ward wear. An ordinary comfortable slipper with a pad of rubber or felt on the heel answers the purpose. See our advertisement columns.

*Army Nursing.*

(214) "Louise," "B. E. S.", and "Red Cross" are anxious for details about army nursing and the training required to enter the service.

Application for admission should in all cases be made to the War Office, Pall Mall, S.W., and candidates must be between 25 and 30 years of age, and have had three years' training in an approved civil general hospital. With regard to the particular inquiry of "Louise," it is seldom desirable to relinquish a certainty for what seems like only a possibility. But the Under Secretary at the War Office will supply all information.

*Daily Nursing Fee.*

(215) Will you kindly tell me what is the correct charge to be made for daily nursing in a small provincial town?—*Inquirer.*

There is no regular charge. Is not the question rather what you can afford to take, what your patients can afford to give, and what demand there is for your services? Your best plan to estimate your charge is to take the sum required by the Queen Victoria's Jubilee Institute for its nurses, namely, £80 a year, or its equivalent, and arrange your fees so as to produce about that sum at first.

*Leprosy.*

(216) Will you tell how I can find out about nursing leprosy; and what is the nearest island set apart for it?—*E. L. C.*

Probably the matron of the female leper wards at Robben Island Infirmary, Cape Colony, could tell you what you want to know. There are many leper asylums, but that on Robben Island is the best known.

*Transvaal.*

(217) In the event of war in the Transvaal I wish to volunteer as nurse. I should be grateful if you will tell me to whom to apply.—*A. C.*

In the event of a war requiring further nursing than that guaranteed by the Army Nursing Service, the Army Nursing Service Reserve (18, Victoria Street, S.W.), would be called out. After that possibly volunteer nurses would be asked for. If properly qualified, however, you could enrol yourself on the Army Nursing Service Reserve.

*The New Treatment.*

(218) I am anxious to take up work in the outdoor treatment of consumption. How can I find out about homes where such treatment is carried out, or to whom should I apply for help? Would it be of any use to put an advertisement in THE HOSPITAL?—*E. B.*

Possibly the best plan would be to advertise in THE HOSPITAL as you suggest. The new homes founded to give this treatment are numerous; but they are not classified as yet.

*Massage.*

(219) What is the best place to go to to learn massage thoroughly? I shall be glad of any information on the subject?—*K. E. G.*

The Society of Trained Masseuses, 12, Buckingham Street, Strand, would be pleased to give you information.

*Drawbacks.*

(220) "C. G.'s" letter is so long that her query is given in abstract. After a month's trial in a small hospital, she was recommended not to continue training. She suffered from a bad sore throat for a time, found the housework part of nursing trying, and has had the misfortune to lose a finger in childhood. Against these drawbacks she sets the fact that she is fond of nursing, and that her family doctor thinks she would make a good nurse. Now she asks us if she should endeavour to continue training.

Has "C. G." realized how very arduous an undertaking nursing is? How much housework must be done? Would she not find a year's training in a children's hospital, with the view to obtain work as child's nurse to a delicate child, equally interesting and more satisfactory?

*One Year.*

(221) Will you please tell me how the one year's special training at the Middlesex ranks in the nursing profession, especially as regards private nursing, and what is the fee required there? I have been told this one year's course is an exception to the general futility of a one-year training. Is this so?—*R.*

As it is impossible to gain the experience and training required by a modern nurse in one year, and as a three years' certificate is an essential qualification for any important work, or for membership in the societies for trained nurses, we do not see why any exception should be made in favour of the training at any one hospital. 2. The fee for lady probationers at the Middlesex is £1 1s. a week for a period of not less than six months.

### TRAVEL NOTES AND QUERIES.

DAVOS (Portsmouth).—Yes, you can do it quite comfortably on the sum named. Your journey will cost each of you £5 11s. 9d. first class, and £3 18s. 6d. second class. This is not return. Six months' board at 6 frs. per day will be £48 each. Then there will be treatment and Kurhaus charges, tips to hotel servants, excursions, library, and many other charges that come flowing in unexpectedly. You can go from Southampton viâ Havre and Zurich. As to clothes, take nothing but woollen of various degrees of thickness; it is very cold in Davos, but the dryness of the atmosphere prevents one suffering from it. I think I should take your tickets from Gaze or Cook, it saves trouble. Take a tea basket, you will find it useful. Hot water bottles and plenty of wraps. Write me again if I can help further.

OBER AMMERGAU (Stevenage).—I must ask you to read our very simple rules; you give me no pseudonym, and can only hope this may meet your eye. Look out next week in the "Mirror" for a further answer; I am just now engaged in getting information on the subject.

